# Morphological and Molecular Analysis of Two Mycophagous Nematodes, *Aphelenchoides bicaudatus* and *A. rutgersi* (Nematoda: Aphelenchoididae) from Florida Strawberry

**DOI:** 10.2478/jofnem-2024-0021

**Published:** 2024-07-05

**Authors:** Clemen J. Oliveira, Sergei A. Subbotin, Johan A. Desager, Paul Dahlin, Silvia Vau, Renato N. Inserra

**Affiliations:** Department of Entomology and Nematology, Gulf Coast Research and Education Center, University of Florida, Wimauma, FL, 33598, USA; Plant Pest Diagnostic Centre, California Department of Food and Agriculture, Sacramento CA, 95832-1448, USA; Centre of Parasitology of A. N. Severtsov Institute of Ecology and Evolution of the Russian Academy of Sciences, Leninskii Prospect 33, Moscow, 117071, Russia; Agroscope, Research Division, Plant Protection, Phytopathology and Zoology in Fruit and Vegetable Production, Wädenswil, Switzerland; Florida Department of Agriculture and Consumer Services, DPI, Nematology Section, P.O. Box 147100, Gainesville FL 32614-7100, USA

**Keywords:** facultative parasitism, foliar nematodes, *Fragaria* × *ananassa*, *Glycine max*, morphology, morphometrics, phylogeny, taxonomy

## Abstract

From 2016 to 2021, nematode surveys in Florida strawberry fields revealed several species of foliar nematodes (*Aphelenchoides* spp.). *Aphelenchoides besseyi sensu stricto* was detected only in 2016 and 2017 on photosynthetic strawberry leaves/buds, but other not well characterized populations of *Aphelenchoides* sp. were found on declining/dessicated leaves. Morphological analyses showed that these samples of *Aphelenchoides* sp. consisted of *A. bicaudatus*, a species detected in Florida for the first time, and *A. rutgersi*, a species previously reported in Florida from the citrus rhizosphere. These two species differed from *A. besseyi* in the shape of their tail terminus: bifurcate in *A. bicaudatus*; mucronate with a ventral thin mucro in *A. rutgersi*; and stellate in *A. besseyi*. One population each of these species was used for morphological and molecular analyses after being reared on *Monilinia fructicola*. Body and tail length differences were observed among Florida *A. bicaudatus* and other populations from the Far East and South Africa. Phylogenetic analyses of the rRNA gene sequences showed that Florida *A. bicaudatus* grouped with those of species from South Korea, Taiwan, and the Netherlands and several other populations listed as *Aphelenchoides* sp. from Brazil, Costa Rica, and Japan, which were considered as representatives of *A. bicaudatus* in this study. Similarly, sequences of Florida *A. rutgersi* grouped with those from environmental samples in Japan and North Carolina, which were listed as *Aphelenchoides* sp. and were considered as representatives of *A. rutgersi* in this study. Photosynthetic strawberry leaf samples were free from both *A. bicaudatus* and *A. rutgersi*, indicating that these two species did not damage strawberry. They were associated with desiccated leaves and/or propagative stolons, usually infected by fungi, confirming that they are mycetophagous under field conditions in this study. Results of soybean leaf inoculation on moist filter paper containing *A. bicaudatus* specimens showed that this species could become phytophagous under artificial conditions. Nematodes penetrated the leaf epidermis and migrated into the mesophyll causing leaf tissue discoloration/necrosis, which remained localized within the infested area. Soybean leaf damage was almost negligible, and no nematode reproduction was observed in the inoculated soybean areas.

Winter strawberry (*Fragaria* × *ananassa* (Duchesne ex Weston) Duchesne ex Rozier) is an economically important commodity in Florida with a cash receipt value of more than $350 million annually (Florida Department of Agriculture, 2013). Nematode parasites of the roots of this crop such as sting nematodes (*Belonolaimus longicaudatus* Rau, 1958) are a major limiting factor of strawberry production in the state. Other plant-parasitic nematodes such as foliar or bud nematodes (*Aphelenchoides* spp.) are largely unknown to Florida strawberry growers. However, infestations of the summer crimp nematode *Aphelenchoides besseyi* Christie, 1942 were prevalent on Florida strawberry from 1930 to the early 1950s. Afterwards, the nematode infestations have been uncommon and not reported until 2016, when they reappeared in a limited number of strawberry operations in Central Florida and damaged strawberry production in 2016 and 2017 (Desaeger and Noling, 2017). In those years, the withdrawal from the market of methyl bromide resulted in foliar and other nematode infestations of propagative strawberry runners produced in nurseries and, consequently, in strawberries plants in the fields (Desaeger and Noling, 2017). These infestations by *A. besseyi* did not reoccur in the following years until the present due to improvement of sanitation practices in nurseries.

Observations conducted in these fields in 2016–2020 indicated that several species of *Aphelenchoides* were present on strawberry plants. These species included *A. besseyi sensu stricto* as defined by Subbotin et al. (2021), found only in 2016 and 2017 on damaged photosynthetic strawberry leaves and buds. Other not well characterized populations of *Aphelenchoides* sp. were found on leaves of declining or desiccated plants. One of these populations with a stellate tail terminus, similar to that of *A. besseyi*, was described by Oliveira et al. (2019) as a new species, *A. pseudogoodeyi*. Two additional populations, one with a bifurcate tail terminus was tentatively identified and reported as *A. bicaudatus* (Imamura, 1931) Filipjev & Schuurmans Stekhoven, 1941 by Oliveira et al. (2018); and another with a thin tail mucro was cataloged as *A. rutgersi* Hooper and Myers, 1971 in the nematode records of the UF Vegetable and Fruit Nematology Lab, University of Florida, GCREC, Wimauma, FL. The tail terminus morphology and morphometric values are an important character for the separation of *Aphelenchoides* species, which were divided into four groups by Shahina (1996). *Aphelenchoides bicaudatus* and *A. rutgersi*, having a tail terminus bifurcate and mucronate with a thin mucro, respectively, belong to group 2, whereas *A. besseyi*, having a stellate tail terminus, belongs to group 3. Thus, these three species can be separated tentatively by the shape of the tail terminus. Morphological characters of *Aphelenchoides* species are variable, making their identification challenging. Comparisons of morphological features and morphometrics with those of described species in original descriptions and compendia are used for these identifications, which are useful in routine diagnostics for nematode management or plant problems. More accurate identification of these species can be obtained by combining the findings of the morphological analysis with those of the molecular character analysis. This approach was used in this study to validate the initial tentative morphological identification of the two Florida populations of *A. bicaudatus* and *A. rutgersi*. These two species have both mycophagous habits and have been reared on *Botrytis cinerea* and *Pyrenochaeta terrestris* (Siddiqi and Taylor, 1966; Hooper and Myers, 1971). It is not known if they are able to feed and reproduce on *Monilinia fructicola*, another fungus commonly used to rear species in the superfamily Aphelenchoidea (Giblin-Davis, 1987; Giblin-Davis et al. 1989, Oliveira et al., 2019; 2022). However, colonization of root tissues of *Phalaenopsis* sp. by *A. bicaudatus* has been reported in Taiwan by Jen et al. (2012), indicating that this species is a facultative parasite under some conditions. After surveying Florida strawberry operations, we found no evidence photosynthetic strawberry leaves had been colonized by *A. bicaudatus*, indicating photosynthetic strawberry leaves are not suitable plant organs to verify facultative parasitism of this species. As an alternative to strawberry, we used soybean (*Glycine max* (L.) Merril), a leguminous plant susceptible to infestations of *Aphelenchoides besseyi* species complex (Favoreto and Mayer, 2017). Soybean seedlings kept in a growth chamber were used for localized inoculation tests conducted by attaching nematode-infested filter paper segments to their leaves. Therefore, this study was conducted to: (i) accurately define the morphological characteristics of these two foliar nematodes; (ii) provide molecular characterization and the phylogenetic relationships of Florida *A. bicaudatus* and *A. rutgersi* with other related species using the 18S rRNA, 28S rRNA, and mitochondrial cytochrome oxidase I (*COI*) gene sequences; (iii) confirm their adaptation to feed on *Monilinia fructicola* (G. Winter), a fungus not used for rearing these two species in the literature; and (iv) determine the ability of *A. bicaudatus* to parasitize soybean seedlings.

## Materials and Methods

*Nematode populations*. In 2017, a survey of foliar nematodes was conducted in a commercial strawberry field of about 10 acres located in Plant City, Florida. The winter strawberry crop of 2016 in this field was destroyed with the herbicide glyphosate by the end of March 2017. Plastic-mulch beds were left intact, as they were to be used again for another strawberry crop in fall 2017. Dead strawberry cv. Florida Radiance plants were pulled from the beds during the summer and left to dry in between the beds. A nematode population tentatively identified as *A. rutgersi* was obtained from leaves of these dead/desiccated strawberry plants at the end of August 2017, and another was identified as *A. bicaudatus* at the beginning of October 2017 (Table 1). Both species were recovered from about 25 g of leaves collected randomly in the field. Leaves were fragmented and incubated in tap water in jars for 12 hr (Young 1954). The number of specimens for each species recovered was 14-56/10 g of incubated leaf tissues and consisted of juveniles and adults mixed with species of *Aphelenchus* sp. and some in the family Rhabditidae. Specimens were assorted and hand-picked with an eyelash and transferred into a Syracuse watch glass containing distilled water. Females morphologically similar and having bifurcate or mucronate tail termini, which are distinct diagnostic characters for their separation, were then selected separately using a stereomicroscope. One to several specimens were then pipetted in a 1-ml drop on two-weeks-old cultures of the fungus *Monilinia fructicola* (G. Winter) growing in potato dextrose agar. This fungus culture has been reported as a conductive medium for rearing species in the superfamily Aphelenchoidea (Giblin-Davis 1987; Giblin-Davis et al. 1989). However, it has not been used to culture *A. bicaudatus* and *A. rutgersi*. A minimum of 10 plates per species was incubated in the dark at 23 ± 3ºC for 23 d, as described by Oliveira et al. (2019) and Oliveira (2022). At the end of the incubation period, specimens that reproduced on fungus migrated on water drops condensed on the lid of the plates and were transferred into watch glasses to be used for morphological and molecular analyses, along with biological studies.

**Table 1.** Populations of *Aphelenchoides* characterized in the present study.

**Species**	**Location**	**Host**	**Sample code**	**GenBank accession number**			**Source**

				**SSU**	**LSU**	***COI***	
*A. bicaudatus*	Wimauma, Florida, USA	*Fragaria* × *ananassa*	N18-1001-3	OK644201	OK644281	OK644199	C. Oliveira
					OK644282		
*A. rutgersi*	Wimauma, Florida, USA	*Fragaria* × *ananassa*	N18-00206	OK644202	OK644283	OK644295	C. Oliveira
				OK644203			

The populations of foliar nematode in the field were monitored from 2018 to 2021 by collecting 43 samples consisting of photosynthetic strawberry leaves showing suspicious foliar nematode damage from six strawberry fields, including those where the initial populations of the two species were collected in desiccated leaves and where infestations of *A. besseyi* occurred in 2017. The photosynthetic leaves were collected to monitor reoccurrence of *A. besseyi* infestations in these fields established with new propagative material. An additional group of 21 samples from desiccated strawberry plants in the same field was also included. These desiccated strawberry leaves were collected in these years to verify persistence of *A. bicaudatus* and *A. rutgersi* in the surveyed fields. A separated group of 183 samples of propagative stolons imported from California, Idaho, and North Carolina in the United Sates, and from Ontario, Nova Scotia, and Quebec in Canada was collected from 2019–2022 and included in this survey to verify introduction of the abovementioned nematodes from outside Florida. All the samples were analyzed for presence of nematodes as described above and were identified only morphologically, since the identity of the original populations was confirmed in the meantime by molecular analyses. The distinct shape of tail terminus of these two species and other morphometrics, such as tail length, were reliable diagnostic characters for their identification in these samples.

*Light microscopic study*. Nematodes in the water suspension from the Petri dishes were picked with an eyelash hair stuck on the end of a mounted needle. Specimens were placed in a drop of water on a glass slide, immobilized by gentle heat, and then mounted in water agar on a slide for measurements and photographs using a modified Esser’s method (Esser, 1986), according to which immobilized specimens were placed and covered with a cover glass on the surface of a water agar block attached to a slide, rather than placing the specimen between two water agar blocks on a slide, as described for this method. A total of 10 and 20 females of *A. bicaudatus* and *A. rutgersi*, respectively, was measured. Seventeen specimens of *A. rutgersi* were also killed by gentle heat, fixed in a solution of 4% formaldehyde + 1% propionic acid and processed in pure glycerin using Seinhorst’s (1966) method and mounted in permanent slides to be used for images and kept at the Nematology Section, Division of Plant Industry, Gainesville, Florida, USA. No permanent slides of *A. bicaudatus* were obtained because the specimens of this species were damaged during the fixation process.

Nematode specimens were examined, measured using a Nikon Labophot (Nikon, Tokyo, Japan) ocular micrometer, and photographed using a compound microscope AXIO Scope A1 equipped with Nomarski interference contrast and an AxioCam ICc5 (Carl Zeiss, Göttigen, Germany). Morphometrics included de Man’s indices and standard measurements suggested by Fortuner (1970) for *Aphelenchoides* species. The morphological and morphometric characters of the Florida population of *A. bicaudatus* with bifurcate tail and the other of *A. rutgersi* with a mucro on tail terminus were compared with those reported for other *Aphelenchoides* species belonging to group 2, according to the classification of *Aphelenchoides* proposed by Shahina (1996), and in the original descriptions of these two species. These characters matched those reported in the abovementioned descriptions, confirming the morphological identity of the two Florida populations of *Aphelenchoides* as *A. bicaudatus* and *A. rutgersi*. Specimens used for molecular character analysis were first identified morphologically and morphometrically.

*DNA extraction, PCR amplification, sequencing, and phylogenetic analysis*. For molecular analyses, nematode DNA was extracted from two to four specimens to obtain the SSU - 18S RNA gene and LSU - 28S rRNA gene and two to six specimens for the *COI* gene of two populations of each species (populations 51, 52 for *A. bicaudatus* and 43 and 48 for *A. rutgersi*) as described by Floyd et al. (2002) and then stored at −20°C until PCR runs. A Bio-Rad thermocycler (Model T100, Bio-Rad, Hercules, California, USA) was used for PCR amplification. A total of 50 µl reaction consisted of 2 µl of extracted DNA; 39.75 µl of nuclease-free water; 5 µl of 10 X ThermoPol reaction buffer; 1 µl of deoxynuxleotide (dNTPs) solution mix (10 mM); 0.25 µl of Taq DNA polymerase (5,000 U/m); and 2 µl of forward and reverse primers (1.0 μg μl−1). Three loci were amplified: the SSU - 18S RNA gene (988-F 5′ – CTC AAA GAT TAA GCC ATG C -3′ / 1912-R – 5′ – TTT ACG GTC AGA ACT AGG G -3′ and 1813-F 5′ – CTG CGT GAG AGG TGA AAT - 3′ / 2646-R 5′ – GCT ACC TTG TTA CGA CTT TT – 3′); the D2-D3 expansion segments of LSU - 28S rRNA gene (D2A – 5′ – ACA AGT ACC GTG AGG GAA AGT – 3′ / D3B – 5′ – CGG AAG GAA CCA GCT ACT A – 3′); and the *COI* gene (COI-F - 5′ – CCT ACT ATG ATT GGT GGT TTT GGT AAT TG – 3′ / COI-R – 5′ – GTA GCA GCA GTA AAA TAA GCA CG – 3′). PCR conditions for amplification were described by Oliveira et al. (2019). PCR products were resolved by electrophoresis at 80 V in 1% agarose gel for 40 min. Amplicons were sent to Genewiz Company (South Plainfield, NJ, USA) for DNA purification and direct sequencing. The nucleotide sequences were obtained by assembling at least two sequences from different specimens within the same population. The new consensus sequences were submitted to the GenBank database under the accession numbers: OK644199 (partial *COI* gene of *A. bicaudatus*), OK644295 (partial *COI* gene of *A. rutgersi*), OK644281 OK64482 (28S rRNA gene of *A. bicaudatus*), OK644283 (28S rRNA gene of *A. rutgersi*), OK644201 (18S rRNA gene of *A. bicaudatus*), and OK644202, OK644203 (18S rRNA gene of *A. rutgersi*).

The newly obtained sequences of partial 18S rRNA, 28S rRNA, and partial *COI* genes were aligned with corresponding published gene sequences (Chizhov et al., 2006; Van Megen et al., 2009; Rybarczyk-Mydłowska et al., 2012; Mueller et al., 2014; de Jesus et al., 2016; Sánchez-Monge et al., 2017; Oliveira et al., 2019; Subbotin et al., 2021) using ClustalX 1.83 (Chenna et al., 2003) with default parameters. Outgroup taxa were selected based on previous publications (Van Megen et al., 2009; de Jesus et al., 2016; Subbotin et al., 2021). Alignments were analyzed with Bayesian inference (BI) using MrBayes 3.1.2 (Ronquist and Huelsenbeck, 2003). The BI analysis for each gene was initiated with a random starting tree and was run with four chains for 1.0 × 10^6^ generations. The Markov chains were sampled at intervals of 100 generations. Two runs were performed for each analysis. After discarding burn-in samples and evaluating convergence, the remaining samples were retained for further analysis. Posterior probabilities (PPs) were given on appropriate clades. Trees were visualized with the TreeView 1.6.6 program (Page, 1996) and drawn with Adobe Illustrator v.10.

*Localized inoculation of a population of A. bicaudatus on soybean leaves*. As stated in the introduction, we conducted inoculations using solely specimens of *A. bicaudatus*. This choice was made due to reports from various authors documenting colonization and reproduction by this species on rice (*Oryza sativa* L.), strawberry, and orchid (*Phalaenopsis* sp.) (Jen et al. 2012; Sánchez-Monge et al., 2015). On the other hand, *A. rutgersi* is currently known only as mycophagous (Hooper and Myers, 1971). The absence of *A. bicaudatus* in the 43 photosynthetic leaf samples from strawberry fields discouraged us from using strawberry for this test. We opted for soybean seedlings because of the glabrous surface of their cotyledons and leaves that facilitates observations of nematode behavior and damaging symptoms induced by nematode feeding activity. Nematodes were inoculated on soybean leaves (cv. Patriot) by applying paper filter pieces containing 300 specimens of the nematode on a selected portion of the upper surface of all leaf blades as described by Riedel (1985) and Oliveira et al. (2019). We used 10 plants to perform two sets of inoculation; each soybean plant received at least three paper filters on different leaves. Five plants were used as a control check. After attaching the infested pieces of filter paper to the leaves, seedlings were enclosed in plastic bags to avoid moisture evaporation and kept in the dark at room temperature for 48 hr. Afterward, seedlings were freed from the plastic bags and filter papers on their leaves, then moved to a greenhouse where they were maintained for 4 wk inside screened cages to avoid damaging arthropod infestation. Seedlings were irrigated with nebulized water twice per day. The inoculated leaves were examined for development of symptoms induced by the nematode three to four times per week using a stereomicroscope. Discolored and necrotic areas on the surface of symptomatic leaves were dissected with a needle to observe and photograph nematode specimens inside the mesophyll.

## Results

***Occurrence of A. bicaudatus and A. rutgersi on strawberry***. The nematological analysis conducted on the 43 samples of photosynthetic strawberry leaves collected between 2018 and 2021 revealed the absence of *A. besseyi, A. bicaudatus*, and *A. rutgersi*. The *A. besseyi* infestations observed on photosynthetic leaves in 2017 did not reoccur in the following years, when new healthy propagative stolons used as resets were produced under improved phytosanitary conditions. A few specimens of *A. bicaudatus* were found in two of the 21 samples of desiccated leaves collected in a strawberry field in Plant City. These findings indicated that *A. bicaudatus* and *A. rutgersi* did not parasitize strawberry under the conditions of the surveyed strawberry fields. These two species were mainly associated with desiccated leaves and/or roots of plants that are usually colonized by fungi. A few specimens of *A. bicaudatus* were detected in three of the 183 samples of imported strawberry transplants with attached soil particles from California and Canada. This detection confirms the behavior of this species, which has been reported in soil associated with many plants, where it feeds on fungi according to Siddiqui (1976), although Jen et al. (2012) in Taiwan recovered *A. bicaudatus* specimens from strawberry buds a month after inoculation and observed eggs in root tissues of inoculated and asymptomatic orchid (*Phalaenopsis* sp.) plants.

### Systematics

*Aphelenchoides bicaudatus* (Imamura, 1931) Filipjev & Schuurmans Stekhoven, 1941.

### Description

This species was described from rice in Japan by Imamura (1931) as *Aphelenchus bicaudatus* and subsequently transferred to the genus *Aphelenchoides* by Filipjev and Schuurmans Stekhoven (1941). Siddiqui and Taylor (1967) and Siddiqui (1976) redescribed this species using a population from turfgrass in Illinois, USA. Herein we provide the morphological characteristics of the Florida population of this nematode collected from desiccated leaves of strawberry.

### Material examined

**Figure 1.**
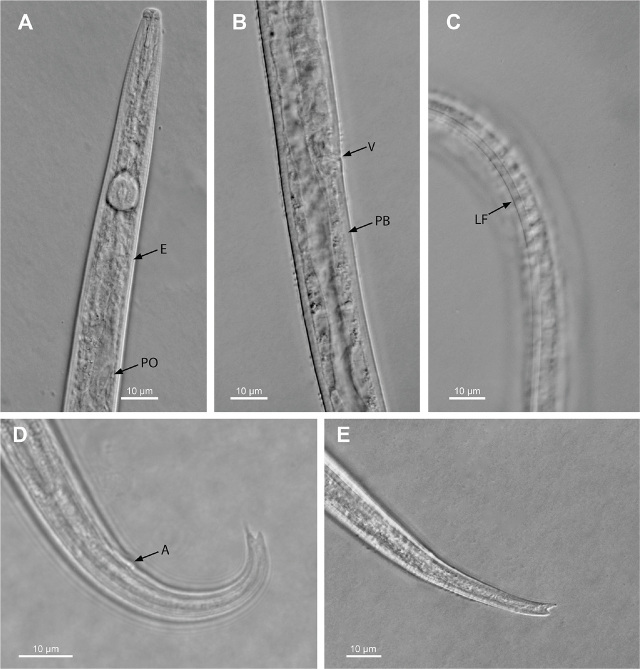
Photomicrographs of *Aphelenchoides bicaudatus* female from Florida. (A) Anterior body showing the (E) excretory pore and the (PO) pharyngeal overlap. (B) Vulvar region showing (V) vulva and (PB) postuterine branch. (C) Lateral field (LF) marked by two outer visible incisures. The inner incisure is not discernible. (D), (E) Posterior body showing (A) anus and shape variations of the bifurcate tail terminus.

Ten live and immobilized Florida females were examined. Morphological features of Florida *A. bicaudatus* are shown in Fig. 1. Morphometrics of this population and other populations from distant geographical areas are listed in Table 2.

**Table 2.** Morphometrics of live females of a Florida population of *Aphelenchoides bicaudatus* from strawberry reared on *Monilinia fructicola* compared to those in the original (Imamura, 1931) and subsequent re-descriptions by Siddiqui & Taylor (1967), Jen et al. (2012), Kim et al. (2016), Israr et al. (2017) and Shokoohi and Moyo (2022).^a^

**Population Substrate**	**Florida** ***Monilinia fructicola***	**Japan** **Paddy field**	**Illinois** ***Pyrenochaeta terrestris***	**Taiwan** ***Alternaria citri***	**South Korea** ***Botrytis cinerea***	**Pakistan** ***Raphanus sativus***	**South Africa** ***Pennisetum clandestinum***

**Reference**	**Present paper N18-01001-3**	**Imamura (1931)**	**Siddiqui and Taylor (1967)**	**Jen et al. (2012)**	**Kim et al. (2016)**	**Israr et al. (2017)**	**Shokoohi and Moyo (2022)**
Character n	10♀♀	18♀♀	50♀♀	50♀♀	7♀♀	2♀♀	8♀♀

L	494.1 ± 32.0			499 ± 67.9	517.9 ± 3.8		455.0 ± 64.5
	(454.8–552.4)	(380.0–470.0)	(410.0–550.0)	(376–637)	(513.6–522.6)	(360.0–360.0)	(409.0–529.0)
A	31.2 ± 3.6			33.0 ± 2.4	28.3 ± 0.5		29.1 ± 2.0
	(27.6–41.1)	(31.3–31.7)	(25–31)	(27.0–38.6)	(27.7–28.8)	(30.1–32.7)	(27.1–31.1)
B	8.0 ± 0.5			9.0 ± 0.7	7.3 ± 0.0		4.6 ± 0.5
	(7.2–9.0)	(6.8–8.4)	(7.3–9.6)	(7.5–10.0)	(7.3–7.4)	(8.8–7.2)	(4.1–5.1)
b’	4.1 ± 0.2	-	-	5.1 ± 0.7	-		-
	(3.7–4.6)			(3.6–7.9)		(5.6–5.8)	
C	13.2 ± 0.9			11.9 ± 0.9	11.3 ± 0.5		16.3 ± 2.0
	(11.9–14.8)	(9.4–12.6)	(9.8–13.7)	(10.1–14.8)	(10.7–11.9)	(11.3–12.0)	(14.1–17.8)
c’	4.4 ± 0.5	-	-	5.4 ± 0.5	4.6 ± 0.1		3.2 ± 0.6
	(3.7–5.3)			(4.1–7.1)	(4.4–4.8)	(2.9–3.7)	(2.6–3.6)
V	68.7 ± 1.5			68.5 ± 1.2	66.0 ± 0.2		68.8 ± 1.6
	(65.8–71.1)	(61.7–90.2)	(65.0–70.0)	(64.9–71.8)	(65.7–66.4)	(66.8–67.2)	(67.0–71.0)
OV	27.8 ± 2.0	-	-	15.2 ± 2.7	-		-
	(23.9–30.9)			(11.0–22.0)		(12.0–12.5)	
Max. body diameter	15.9 ± 1.3	-	-	-	-		15.6 ± 1.5
	(13.5–17.8)					(25.0–26.2)	(14.0–17.0)
Body diameter at anus	8.6 ± 0.8	-	-	-	-		8.8 ± 0.7
	(7.2–9.9)					(11.8)	(8.0–9.0)
Anterior genital tract length	136.9 ± 10.4	-	-	-	-		179.3 ± 18.0
	(121.7–150.1)					(84.0–95.0)	(167.0–200.0)
Lip region width	4.9 ± 0.3	-	-	-			5.7 ± 0.1
	(4.5–5.5)				(5.0–5.2)	(4.0–4.3)	(5.6–5.8)
Lip region height	2.8 ± 0.2	-	-	-			2.8 ± 0.9
	(2.4–3.0)				(2.5–2.9)	(2.0–2.0)	(2.0–4.0)
Stylet length	10.9 ± 0.4	-		10.4 ± 0.6	11.2 ± 0.5		11.1 ± 1.7
	(10.3–11.8)		(10–12)	(9.0–12.0)	(10.4–11.7)	(10.0–11.0)	(10.0–13.0)
Stylet cone	5.1 ± 0.3	-	-	-	-	-	4.7 ±0.3
	(4.5–5.8)						(4.5–5.0)
Stylet knob height	1.4 ± 0.2	-	-	-	-	-	-
	(1.2-1.7)						
Stylet knob width	1.9 ± 0.3	-	-	-	-	-	-
	(1.2–2.3)						
Median bulb length	12.1 ± 0.7	-	-	-			11.2 ± 0.7
	(10.9–13.3)				(12.8–12.9)	(10.0–10.0)	(11.0–12.0)
Median bulb width	9.6 ± 0.8	-	-	-			9.0 ±0.8
	(7.9–11.1)				(8.6–8.9)	(7.0–8.0)	(8.5–10.0)
Median bulb valve length	4.1 ± 0.4	-	-	-	-	-	-
	(3.6–4.9)						
Median bulb valve width	2.9 ± 0.2	-	-	-	-	-	-
	(2.5–3.2)						
Pharynx length	62.1 ± 2.7	-	-	-	-		88.3 ± 11.2
	(59.0–68.7)					(90.0–92.0)	(76.0–98.0)
Pharyngeal overlap	57.0 ± 2.1	-		-	-	-	-
	(53.0–59.7)		(50.0–75.0)				
Ant. end to pharyngeal gland lobe	118.8 ± 3.3	-	-	-	-	-	-
	(4.5–5.8)						
Anterior end to excretory pore	66.9 ± 3.0	-	-	-	-		61.3 ± 3.2
	(62.3–71.2)					(50.0–51.0)	(60.0–65.0)
Postuterine sac (PUS)	22.3 ± 1.9	-	-	-	-		47.0 ± 2.6
	(18.0–24.2)					(22.0–24.0)	(45.0–50.0)
Vulva anus distance (VA)	123.5 ± 10.3	-	-	-	-		-
	(105.9–137.6)					(84.0–85.0)	
Ant. end to vulva	349.3 ± 22.3	-	-	-	-		-
	(311.0–392.7)					(242.0–248.0)	
Post end to vulva	144.8 ± 26.5	-	-	-	-	-	-
	(89.3–170.4)						
Tail length	37.4 ± 0.8	-			45.9 ± 2.5		28.0 ± 3.6
	(36.6–38.6)		(41.8)	(34–42)	(43.0–48.8)	(30.0–31.0)	(24.0–31.0)
Body width at vulva (BWV)	14.5 ± 0.8	-	-	-	-	-	-
	(13.1–15.8)						
PUS/L	4.5 ± 0.4	-	-	-	-		-
	(3.7–5.2)					(6.1–6.6)	
PUS/BWV	1.6 ± 0.2	-	-	-	-	-	-
	(1.1–1.8)						
PUS/VA	18.1 ± 1.1	-	-	18.9 ± 4.5	-		-
	(16.0–20.5)			(9.2–33.8)		(22.4–24.0)	
Lateral field width	2.7 ± 0.4	-	-		-	-	-
	(2.1–3.4)			(2.2)			

a All measurements are given in micrometers except the ratios a, b, b’, c, c’ and the percentages V, OV, PUS/L, and PUS/VA. Mean ± SD (range).

### Adult

The adult stages that were found in our strawberry population consisted of females. No males were detected.

***Female:*** Body straight, attenuated anteriorly and narrowing posteriorly with a slightly curved or arcuate tail. Body marked by faint annuli. Lateral field 2.7 µm wide and marked by three incisures. The inner incisure faint and not well discerniable with the microscope we used. Head slightly set off. Stylet 10.3–11.8 µm long and with evident knobs. Pharyngeal middle bulb round 9.6 µm wide and with a pronounced valve, 3 *µ*m long. Pharyngeal gland elongated and overlapping the intestine dorsally. Excretory pore opening at level of the nerve ring. Hemizonid not seen. Ovary single occupying 26% of body length. Vulva a transverse slit. Post uterine branch 22 µm long. Tail straight or arcuate ending in a bifurcate tip.

### Distribution

*Aphelenchoides bicaudatus* has been reported from distant geographical areas other than Japan and Illinois (USA). These regions include Australia, Brunei, France (Siddiqui, 1976) Pakistan (Israr et al., 2017), South Africa (Shokoohi and Moyo, 2022), South Korea (Kim et., 2016), and Taiwan (Jen et al., 2012) and, from this study, Brazil, and Costa Rica.

### Remarks

Our Florida population fits the characteristics of other populations from the distant geographical areas listed above. However, some morphometrical differences were observed in females of Florida strawberry population, which have a shorter tail and greater values of ratio c than those reported by Siddiqui & Taylor (1967) (36.6–38.6 versus 41.8 *μ*m and 11.9–14.8 versus 9.8–13.7, respectively), and also a population reported by Kim et al. (2016) from South Korea (36.6–38.6 versus 43.0–48.8 *μ*m and 11.9–14.8 versus 10.7–11.9, respectively). Tail length and c values of the Florida population were, respectively, greater and smaller than those reported for populations from Pakistan (36.6–38.6 versus 30.0–31.0 *μ*m and 11.9–14.8 versus 11.3–12) by Israr et al. (2017) and South Africa (36.6–38.6 versus 24.0–31.0 *μ*m and 11.9–14.8 versus 14.1–17.8) by Shokoohi and Moyo (2022). Florida *A. bicaudatus* females have a lateral field marked by two distinct outer incisures and an inner and faint incisure not well discernible with the optical system that was used. Three distinct incisures in the lateral field were reported in a population from Illinois used for the redescription of this species and in another from Taiwan. Two incisures were observed in populations from Pakistan, South Africa, and South Korea. These morphometric variations were not reflected by the results of the phylogenetic analysis using the two genes mentioned above.

*Aphelenchoides rutgersi* Hooper and Myers, 1971.

### Description

This species was found by Hooper and Myers (1971) in a soil sample collected from the rhizosphere of citrus from central Florida. These authors reared this population on two fungi (*Pyrenochaeta terrestris* and *Botrytis cynerea*) growing in media in Petri dishes and used the specimens from these fungal cultures to describe this species. Herein we provide details on morphological characteristics of the population of this nematode collected from desiccated leaves of strawberry.

### Material examined

Twenty live and immobilized females and two males.

**Figure 2.**
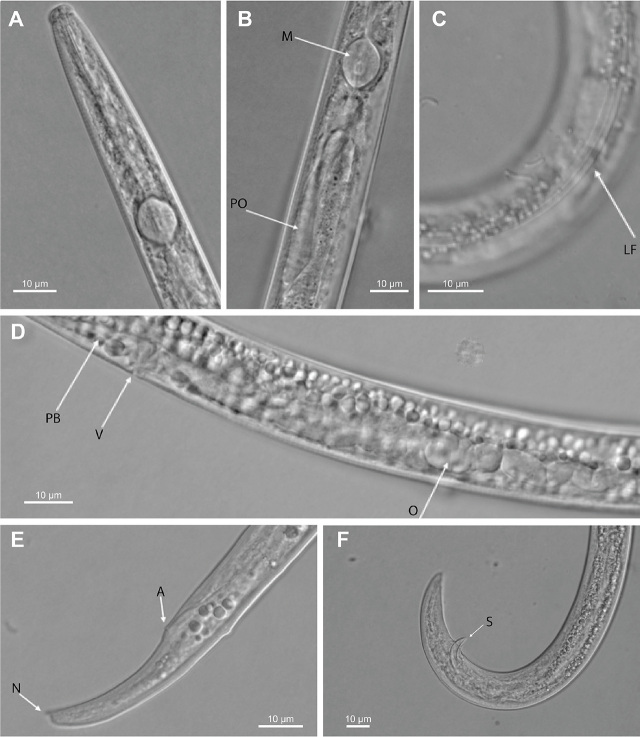
Photomicrographs of *Aphelenchoides rutgersi* female and male from Florida. (A) Female anterior body. (B) Pharyngeal region of female showing (M) median bulb and (PO) pharyngeal overlap. (C) Lateral field (LF) marked by three incisures. (D) Vulvar region showing (O) ovary with oocytes, (V) vulva, and portion of the (PB) post-vulvar uterine branch. (E) Posterior body of female showing (A) anus (A) and (N) the tail terminus with a ventral fine mucro. (F): Posterior body of male showing (S) spicules.

Measurements and morphological features of *A. rutgersi* from strawberry are shown in Tables 3, 4, and Fig. 2.

**Table 3.** Morphometrics of live females of a Florida populations of *Aphelenchoides rutgersi* from strawberry and reared on *Monilinia fructicola* compared to those in the original description by Hooper and Myers (1971) and populations from Pakistan and South Africa reported by Erum and Shahina (2010) and Girgan et al. (2018), respectively.^a^

**Population Substrate**	**Florida** ***Monilinia fructicola***	**Florida** ***Pyrenochaeta terrestris***	**Florida** ***Botrytis cinerea* for 2 weeks**	**Florida** ***B. cinerea* for 7 weeks**	**Pakistan** ***Triticum* sp.**	**South Africa** ***Aristida canescens* seeds**

**Reference**	**Present study (N18-00206)**	**Hooper and Myers (1971)**	**Hooper and Myers (1971)**	**Hooper and Myers (1971)**	**Erum and Shahina (2010)**	**Girgan at al. (2018)**
Character n	20♀♀	20♀♀	20♀♀	20♀♀	12♀♀	3♀♀

L	507.6 ± 71.0	535.0 ± 20	480.0 ± 25	360.0 ± 20	440 ± 30	556.0 ± 22.6
	(371.2–614.7)	(500.0–570.0)	(430.0–530.0)	(320.0–405.0)	(370.0–500.0)	(542.0–582.0)
A	29 ± 2	27.0 ± 1.8	25.0 ± 1.7	27.0 ± 1.7	26.7 ± 1.4	34.1 ± 0.8
	(25.8–33.5)	(23–30)	(23–29)	(24–31)	(24.0–31.7)	(33.5–34.9)
B	8.4 ± 1.0	10.7 ± 0.4	9.3 ± 0.4	7.60 ± 0.16	5.7 ± 1.3	4.6 ± 0.2
	(6.5–10.0)	(9.7–11.6)	(8.3–10.1)	(6.7–8.9)	(4.4–8.4)	(4.4–4.8)
b’	4.0 ± 0.6	5.0 ± 0.5	4.1 ± 0.4	3.5 ± 0.3	4.4 ± 0.8	-
	(3.0–5.2)	(4.2–6.2)	(3.6–4.6)	(3.1–4.0)	(4.1–5.0)	
C	15.4 ± 1.7	16.9 ± 0.6	16.3 ± 1.0	14.1 ± 0.8	8.0 ±0.4	15.8 ± 0.5
	(12.9–18.9)	(15.9–18.1)	(15.0–18.7)	(12.4–15.5)	(7.2–8.6)	(15.5–16.4)
c’	3.4 ± 0.4	3.0 ± 0.2	2.9 ± 0.1	3.3 ± 0.2	5.7 ± 0.4	3.6 ± 0.3
	(2.5–4.0)	(2.6–3.4)	(2.6–3.2)	(2.8–4.0)	(4.8–6.4)	(3.3–3.9)
V	70.3 ± 1.0	71.0 ± 1.0	72.0 ± 0.9	70.0 ± 0.7	64.5 ± 0.9	69.0 ± 0.8
	(68.6–72.0)	(69.0–72.0)	(70.0–74.0)	(69.0–72.0)	(63.3–65.5)	(69.0–70.0)
Max. body diameter	17.6 ± 3.1	-	-	-	-	16.0 ± 0.9
	(12.8–22.7)					(16.0–17.0)
Body diameter at anus or cloacal opening	10.0 ± 1.4	-	-	-	-	10.0 ± 0.9
	(7.4–13.3)					(9.0–11.0)
Body diameter at vulva (BDV)	18.1 ± 2.3	-	-	-	-	16.0 ± 0.8
	(15–23)					(15.017.0)
OV or Testis/L	24.8 ± 6.4	60.0 ± 7.1	50.0 ± 4.6	24.0 ± 2.8	-	-
	(15.4–40.9)	(50–77)	(42–60)	(18–29)		
Anterior genital tract length	127.2 ± 44.0	-	-	-	-	220.0 ± 5.6
	(70.0–242.5)					(215.0–226.0)
Lip region width	6.3 ± 0.5	-	-	-		5.6 ± 0.4
	(5.0–6.9)				(4.8–5.6)	(5.0–6.0)
Lip region height	2.9 ± 0.2	-	-	-		3.2 ± 0.5
	(2.5–3.0)				(2.4–3.2)	(3.0–4.0)
Stylet length	11.0 ± 0.5		-	-	10.7 ± 0.6	11.0 ± 0.4
	(10.1–11.9)	(10.0)			(10.0–12.0)	(11.0–12.0)
Stylet cone	5.4 ± 0.4	-	-	-	-	-
	(4.8–6.0)					
Stylet knob height	1.5 ± 0.2	-	-	-	-	-
	(1.2–1.9)					
Stylet knob width	2.3 ± 0.3	-	-	-	-	-
	(1.9-2.8)					
Median bulb length	13.7 ± 0.7	-	-	-		12.0 ±0.5
	(12.3–14.9)				(12.8–13.6)	(12.0–13.0)
Median bulb width	10.3 ± 0.7	-	-	-		10.0 ± 0.5
	(9.0-11.9)				(9.6–10.4)	(9.0–10.0)
Median bulb valve length	4.0 ± 0.4	-	-	-	-	-
	(3.1–4.5)					
Median bulb valve width	3.0 ± 0.2	-	-	-	-	-
	(2.5–3.4)					
Pharynx length	60.4 ± 2.1	44 ± 2	46 ± 2	27.0 ± 1.7	-	
	(54.4–66.3)	(42–47)	(42–49)	(24–31)		(58.0)
Pharyngeal overlap	67.5 ± 12.5	-	-	-	-	-
	(45.5–95.8)					
Ant. end to pharyngeal gland lobe	127.8 ± 13.7	-	-	-	-	122.0 ± 1.5
	(99.9–162.1)					(121.0–124.0)
Anterior end to excretory pore	66.6 ± 5.4	-	-	-	-	76.0 ±1.9
	(58.4–76.2)					(75.0–76.0)
Postuterine sac	26.1 ± 4.5	-	-	-	42.6 ±2.2	40.0 ± 4.1
	(15.8–41.0)				(36.0–55.0)	(37.0–43.0)
Vulva anus distance	117.7 ± 19.2	-	-	-	-	-
	(76.2–149.4)					
Ant. end to vulva	356.9 ± 50.3	-	-	-	386.0 ± 13.6	-
	(266.3–437.5)				(374.0 401.0)	
Post end to vulva	150.7 ± 21.7	-	-	-	-	-
	(104.9–191.9)					
Tail length	33.0 ± 3.6		-	-	35.0 ± 2.1	-
	(26.7–42.5)	(30.5)			(33.0–37.0)	
Spermatheca length	14.1 ± 3.9	-	-	-	-	-
	(9–21)					
Spermatheca width	9.9 ± 1.6	-	-	-	-	-
	(8–13)					
PUS/VA	22.1 ± 3.8	-	-	-	-	-
	(16.1–27.5)					
Lateral field width	3.0 ± 0.2	-	-	-	-	-
	(2.5–3.4)					
PUS/L	5.1 ± 0.8	-	-	-	-	-
	(3.8–6.4)					
PUS/BDV	1.5 ± 0.2	-	-	-	-	-
	(1.0–1.9)					
Spikes	1	1	1	-	1	-

a All measurements are in micrometers and in the form: mean ± SD (range).

**Table 4.** Morphometrics of live males of a Florida population of *Aphelenchoides rutgersi* from strawberry reared on *Monilinia fructicola* compared to those in the original description by Hooper and Myers (1971) and a population from Pakistan described by Erum and Shahina (2010).^a^

**Population Substrate**	**Florida** ***Monilinia fructicola***	**Florida** ***Botrytis cinerea***	**Pakistan** ***Triticum* sp.**

**Reference**	**Present study N18-00206**	**Hooper and Myers (1971)**	**Erum and Shahina (2010)**
Character n	2♂♂	20♂♂	7♂♂

L	538.8 ± 20.5	440.0 ± 35	400.0 ± 30.0
	(518.3−559.2)	(380.0−490.0)	(360.0−470.0)
A	29.7 ± 0.3	29.0 ± 2.8	28.7 ± 2.5
	(29.4−30.0)	(21−32)	(24.0−31.7)
B	8.9 ± 0.4	8.7 ± 0.9	5.3 ± 1.3
	(8.5−9.3)	(7.1−11.0)	(3.9−7.2)
b’	4.0 ± 0.1	4.0 ± 0.1	4.5 ± 1.0
	(4.0−4.1)	(3.6−4.4)	(4.2−5.5)
C	15.9 ± 1.2	14.3 ± 0.8	13.7 ± 1.6
	(14.7−17.2)	(13.0−15.4)	(10.6−16.0)
c’	2.6 ± 0.3	2.9 ± 0.2	2.7 ± 0.3
	(2.3−2.9)	(2.4−3.3)	(2.4−3.1)
Max. body diameter	18.2 ± 0.9	-	-
	(17.3−19.0)		
Body diameter at cloacal opening	13 ± 1	-	-
	(12−14)		
Testis	240.5 ± 8.5	-	-
	(232−249)		
Testis/L%	44.7 ± 0.1	72.0 ± 6.2	-
	(44.5−44.8)	(58.0−78.0)	
Lip region width	6.5 ± 0.2	-	-
	(6.3−6.6)		
Lip region height	3	-	-
Stylet length	11.9	10.0 ± 1 (9.5−11.0)	10.6 ± 0.4
			(10.0−11.2)
Stylet cone	6	-	-
	(5.9−6.0)		
Stylet knob width	2.4 ± 0.1	-	-
	(2.3−2.4)		
Stylet knob height	1.8 ± 0.1	-	-
	(1.7−1.8)		
Median bulb width	10.0 ± 0.5	-	-
	(9.5−10.5)		
Median bulb length	14.2 ± 0.7	-	-
	(13.5−14.8)		
Median bulb valve length	4.5	-	-
Median bulb s valve width	3	-	-
Pharynx length	60.6 ± 0.2	45 ± 3	-
	(60.4−60.8)	(38.0−51.0)	
Pharyngeal overlap	72.8 ± 3.5	-	-
	(69.3−76.2)		
Ant. end to pharyngeal gland lobe	133.7 ± 2.9	-	-
	(130.8−136.6)		
Anterior end to excretory pore	67.8 ± 3.5	-	-
	(64.3−71.2)		
Tail length	33.9 ± 1.3	-	-
	(32.6−35.2)		
Spicule length	18.2 ± 0.6	15.5 ± 1.0	14.2 ± 1.7
	(16.6−19.8)	(14−17)	(13.0−17.0)
Lateral field width	3	-	-

a All measurements are given in micrometers, except for the ratios a,b, b’,c and c’ and the percent values Testis/L%. Mean ± SD (range).

### Adult

***Female:*** Body straight tapering slightly anteriorly. Head slightly offset. Stylet 11 *μ*m long, with small knobs. Pharynx with the characteristics of the genus. Pharyngeal medial bulb spheroid with the valve located in central or postmedian position. Pharyngeal glands forming a distinct lobe that overlaps the intestine dorsally. Lateral field 3 *μ*m wide and marked by three incisures. Excretory pore at level of nerve ring. Hemizonid not observed. Ovary single with oocytes in a straight row. Vulva a transverse slit. Postuterine branch short, 5 *μ*m in length. Tail with a slight curvature ending in a short ventral mucro.

***Male:*** Body similar to that of female. Tail arcuate, ending in a short mucro. Spicules 18 *μ*m long.

### Distribution

This species was described in Florida (USA) and reported in Bulgaria (Katalan-Gateva and Budurova, 1976), Pakistan (Erum and Shahina, 2010), and South Africa (Girgan et al., 2018), and Japan (this study).

### Remarks

The morphology of the female specimens of our population reared on the fungus *M. fructicola* for 30 d was variable. Numerous females with small body size were mixed with a few specimens having a large body. Many allometric characters were also variable in our study because of this body size variability. This variability in the morphology of this species was observed among the populations reared on the two fungi and for a different length of time in the original description of this species. However, the range of morphometric characters of our population and those of the original description overlapped and matched those of a population from South Africa. The average length of stylet of our population was slightly (10%) greater than that of the stylet reported in the original description and was like that of populations from Pakistan and South Africa. The specimens we studied had also a longer pharynx. The other characters did not differ. The lateral field was marked by three incisures, with the inner one thicker than the outer ones. The genital tract of our females consisted of an ovary that did not reach the end of the esophageal glands and did contain large oocytes. No spermatheca was observed in it. The postuterine sac was variable in length and shape, elongated and tapered in some specimens but sack-like in others. The tail was slightly curved ventrally and with a ventral terminal mucro variable in length and devoid of processes, as reported in the original description. The terminal mucro in our population was more ventrally located than in the original description and the Pakistan and South Africa populations. Females of the population from Pakistan had a longer tail (52.0 versus 30.5, 33.0 and 35.0 *µ*m) and a more anterior position calculated as percentage of the total body length (63.0–65.5 versus 69.0–74.0, 68.6–72.0 and 69.0–70.0) of the vulva than those in the original description and populations from Florida and South Africa.

Male body and spicules in the original description were 380–490 and 14–17 *µ*m long, respectively. The two male specimens in our population had larger bodies than those in the original description and the population from Pakistan (518–559 *µ*m versus 380–490 and 360–470 *µ*m). They had also slightly longer stylet and pharynx. The length of the spicules was also slightly longer than those in the original and Pakistan populations (16.6–19.8 versus 14.0–17.0 and 13.0–17.0 *µ*m). In the Florida population, males might play a minor role in the reproduction of this species, since the females we examined did not have functional spermatheca and presence of spermatozoa in the genital tract.

Molecular characterization and phylogenetic relationships of Florida *Aphelenchoides bicaudatus* and *A. rutgersi* with other species.

### 18S rRNA gene

**Figure 3.**
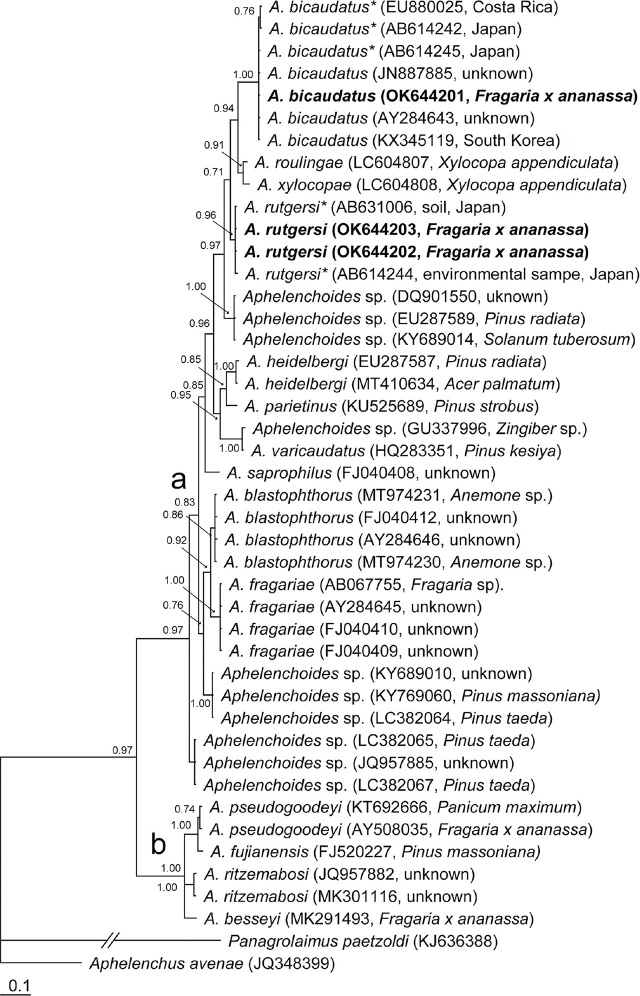
Phylogenetic relationships of *A. bicaudatus* and *A. rutgersi* with some *Aphelenchoides* species: Bayesian 50% majority rule consensus tree from two runs as inferred from analysis of the 18S rRNA gene sequence alignment under the GTR + I + G model. Posterior probabilities equal to, or more than, 0.7 are given for appropriate clades. New sequences are indicated in bold letters. * - identified as *Aphelenchoides* sp. in the GenBank.

The 18S rRNA gene alignment contained 46 sequences, including one new sequence of Florida *A. bicaudatus* and two new sequences of *A. rutgersi,* and was 631 bp in length. Florida *A. bicaudatus* and *A. rutgersi* sequences belonged to the *Aphelenchoides* clade “a” (Fig. 3). Florida *A. bicaudatus* sequences clustered in a highly supported clade (PP=1.0) with those of other populations of this species from South Korea, Taiwan, the Netherlands, and three *Aphelenchoides* sp. sequences from Japan (Kanzaki and Mizukubo, unpublished) and Costa Rica (Powers et al., 2009) and considered here as representatives of *A. bicaudatus.* Intraspecific variation for *A. bicaudatus* was 0%–0.1% (0–1 bp). The two sequences of Florida *A. rutgersi* were identical and clustered in a clade with a high support (PP=1.0) with two sequences of *Aphelenchoides* sp. obtained from environmental samples in Japan (Kanzaki and Mizukubo, unpublished) and considered here as representatives of *A. rutgersi*. Intraspecific variation for *A. rutgersi* was 0%–0.1% (0–1 bp).

### 28S rRNA gene

**Figure 4.**
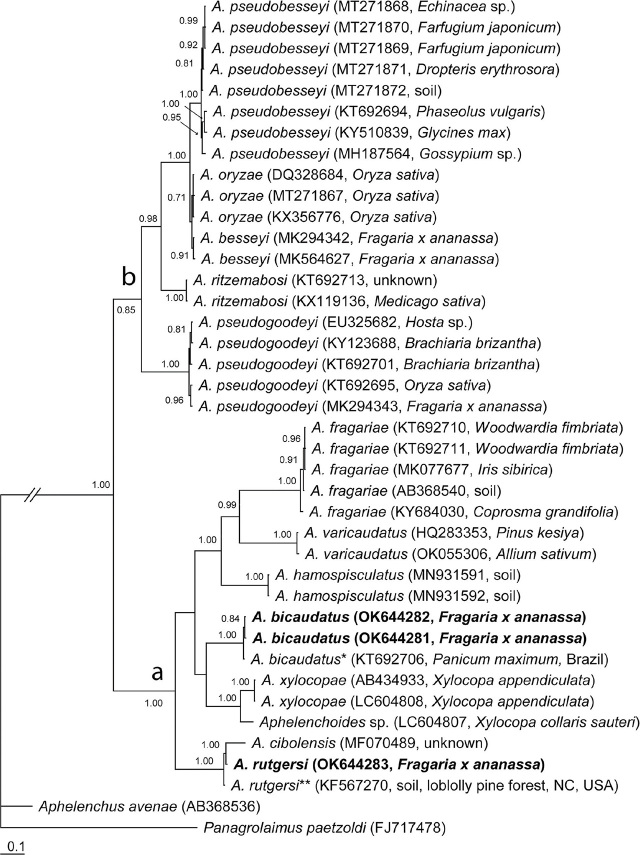
Phylogenetic relationships of *A. bicaudatus* and *A. rutgersi* with some *Aphelenchoides* species: Bayesian 50% majority rule consensus tree from two runs as inferred from analysis of the D2-D3 of 28S rRNA gene sequence alignment under the GTR + I + G model. Posterior probabilities equal to, or more than, 0.7 are given for appropriate clades. New sequences are indicated in bold letters. * - identified as *Aphelenchoides* sp. in the GenBank. ** - identified as an uncultured fungus in the GenBank.

The 28S rRNA gene alignment contained 40 sequences, including two new sequences for Florida *A. bicaudatus* and a new sequence for Florida *A. rutgersi*, and was 627 bp in length. The phylogenetic relationships of Florida *A. bicaudatus* and *A. rutgersi* within selected species of the genus *Aphelenchoides* are given in Fig. 4. The two sequences of Florida *A. bicaudatus* were identical and clustered in a clade with a high support (PP=1.0) with that of an *Aphelenchoides* sp. from *Panicum maximum* in Brazil (de Jesus et al., 2016), which was considered here as representative of *A. bicaudatus.* Intraspecific variation for *A. bicaudatus* was 0.3%–0.9% (2–5 bp). The sequence of the Florida *Aphelenchoides rutgersi* formed a highly supported clade (PP=1.0) with an *Aphelenchoides* sp. sequence obtained from an environmental sample from North Carolina (Mueller et al., 2014), which was considered here as a sequence of *A. rutgersi,* and with a sequence of a population identified as *A. cibolensis* Riffle, 1970 by Heydari et al. (unpublished). Intraspecific variation for *A. rutgersi* was 1.0% (1\4 bp).

### COI gene

**Figure 5.**
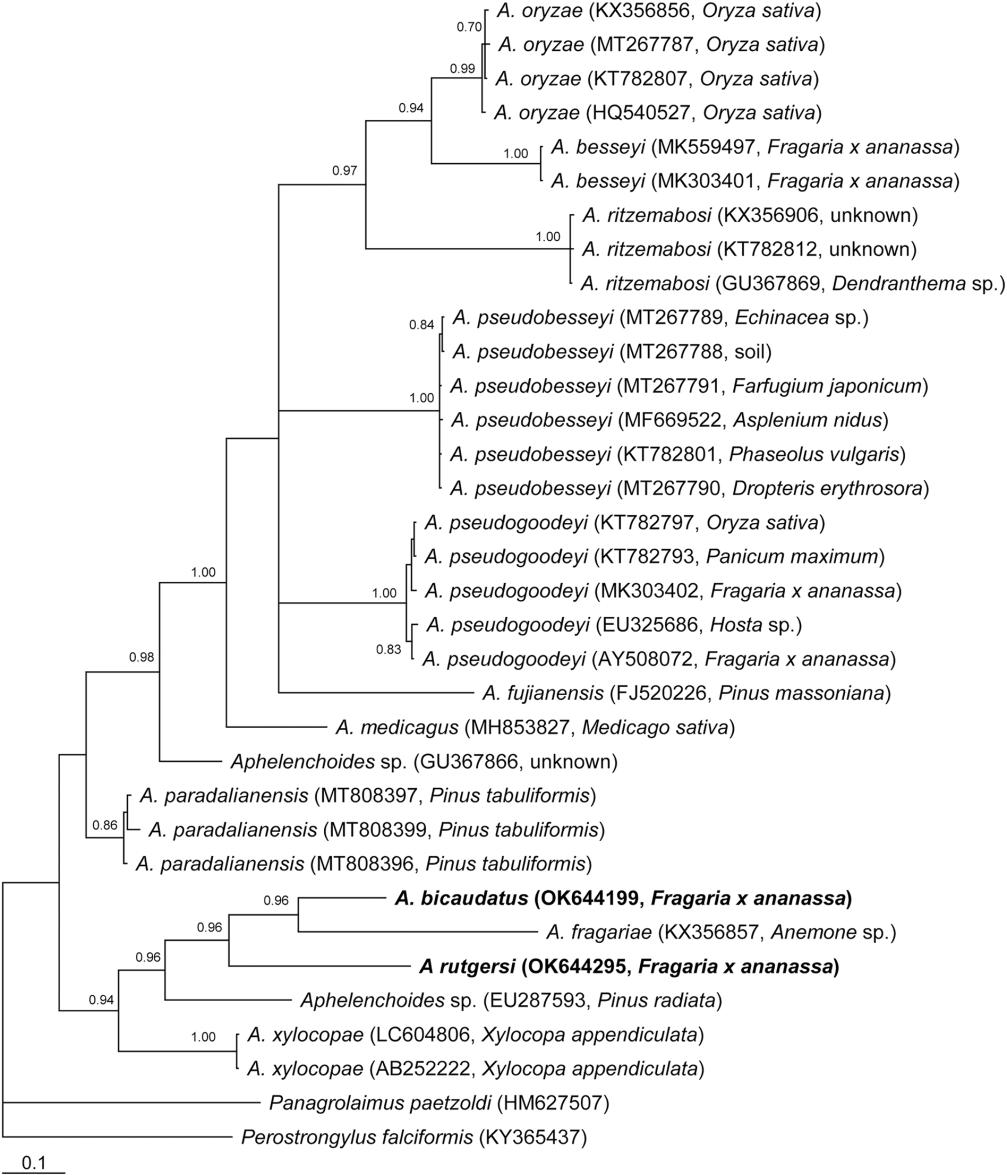
Phylogenetic relationships of *A. bicaudatus* and *A. rutgersi* with some *Aphelenchoides* species: Bayesian 50% majority rule consensus tree from two runs as inferred from analysis of the *COI* gene sequence alignment under the GTR + I + G model. Posterior probabilities equal to, or more than, 0.7 are given for appropriate clades. New sequences are indicated in bold letters.

The *COI* of mtDNA alignment contained 34 sequences of *Aphelenchoides* species, including a new sequence for Florida *A. bicaudatus* and a new sequence for Florida *A. rutgersi* from strawberry and was 527 bp in length. The phylogenetic relationships of Florida *A. bicaudatus* and *A. rutgersi* within selected species of the genus *Aphelenchoides* are given in Fig. 5. The new sequences of Florida *A. bicaudatus* and *A. rutgersi* clustered with *A. fragariae* (Ritzema Bos, 1890) Christie, 1932. Sequences of Florida *A. bicaudatus* and *A. rutgersi* differed in 13.8% (90 bp).

**Figure 6.**
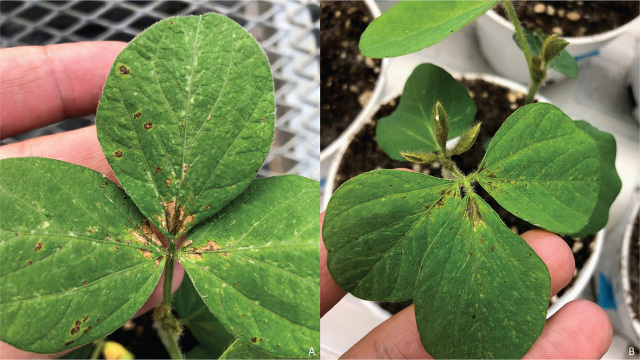
Symptoms induced by *Aphelenchoides bicaudatus* on soybean leaves. (A) Seedling showing discoloration and necrosis on the upper surface of the blade adjacent to the petiole 3 d after inoculation of the nematode using pieces of filter paper attached to the leaf Blade. (B) Closeup of the lesion delimited by the veins of the leaflet showing desiccated areas and dark tissues along the veins 14 d after the inoculation of the nematode.

**Figure 7.**
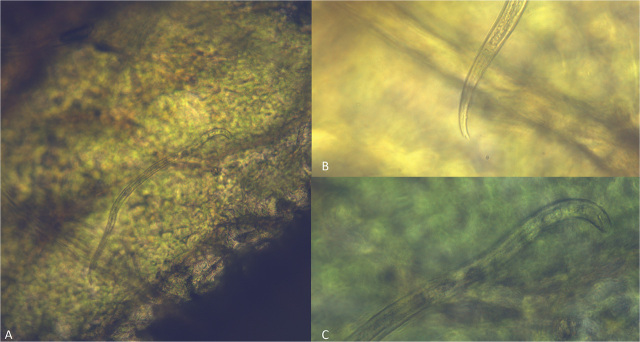
*Aphelenchoides bicaudatus* inside soybean leaf tissues. (A) A nematode specimen tunneling the mesophyll 7 d after the inoculation of 300 specimens delivered with a piece of filter paper attached to the leaf blade. (B) Posterior body of a specimen showing the bifurcate tail terminus. (C) Anterior body of a specimen showing the stylet. (Scale bar = 24 *μ*m).

***Localization inoculation of a population of A. bicaudatus on soybean leaves.*** This experiment was conducted with *A. bicaudatus* only. The inoculation test of soybean leaves with pieces of filter paper containing specimens of this species resulted in discoloration and small necrotic areas on the portion of the leaves in contact with the nematode-infested filter paper. The symptoms appeared 3 d after inoculation and became more evident on the 14th day, when the base of the upper blades in contact with the petioles became necrotic (Figs. 6,7). Nematodes inside the necrotic spots became visible by tearing the leaf epidermis with a needle at the level of the necrotic areas, exposing the nematodes that were visible using a stereo microscope inside the spongy parenchyma and palisade tissues. The number of nematodes inside the necrotic areas varied from one to two (Figs. 7 A–C). No expansion of these lesions on the leaf blade was observed after leaving the plants in the greenhouse for two additional weeks or 38 d. The lack of progression of the symptoms in the leaves almost 40 days after inoculation indicated that the nematode did not increase its populations and failed to colonize the soybean leaves.

## Discussion

The present work is part of a long-term project initiated in 2016 on foliar nematodes associated with the leaves of strawberry in commercial operations in central Florida. In this study, additional species of foliar nematodes were detected confirming the great diversity of species associated with the above ground part of this industrial crop. The detections of *A. bicaudatus* and *A. rutgersi* on Florida desiccated strawberry leaves are the first and second report in Florida for the former and latter species, respectively. *Aphelenchoides rutgersi* was found and described 40 yr ago in a citrus orchard in the vicinity (48 km east) of the strawberry fields. No *A. besseyi* was observed in our survey in 2018–2021 after the infestations of this nematode that occurred in 2017. This infestation was probably caused by North Carolina transplants infested with *A. besseyi*. One probable reason for the disappearance of this nematode in 2018 and following years was the adoption of improved sanitation practices in North Carolina strawberry nurseries where production fields were relocated. Unfavorable weather conditions may have, also, avoided reinfestations in Florida fumigated fields.

Our observations on the behavior of *A. bicaudatus* and *A. rutgersi* on strawberry indicated that these species were not parasites of this crop under field conditions. Both species, as in the case of the phytoparasite *A. besseyi* (Oliveira et al., 2022), reproduced in cultures of the fungus *M. fructicola,* a new fungus host for both species confirming their mycetophagous habits. The morphological differences among our populations of *A. bicaudatus* and *A. rutgersi* and other populations of these species involved mainly allometric characters such as tail length. The nonallometric characters, such as stylet length, which are not influenced by body size, did not differ among these populations.

The phylogenetic analysis of Florida *A. bicaudatus* using rRNA gene sequences showed that this species grouped with other populations of this species from distant geographical areas despite their morphological variability. Florida *A. rutgersi* rRNA gene sequences clustered together with other *Aphelenchoides* sequences from Japan and North Carolina, which were identified here as representatives of this species. Sequences of both species belonged to clade “a” of *Aphelenchoides* as it has been defined by Ryss et al. (2013), which includes the plant parasitic *Aphelenchoides fragariae*. *Aphelenchoides* species parasitizing strawberry were found in both clades (a and b).

The results of inoculation of soybean leaves with moist filter paper containing *A. bicaudatus* specimens indicate that this species can become phytophagous in artificial infestation conditions. Nematode infestation caused discoloration and necrosis of the leaf tissues, which remained localized and did not expand. Damage to soybean leaves was almost negligible, confirming reports of colonization and reproduction without damaging symptoms of other populations of this species on rice (*Oryza sativa* L.) and roots of *Phalaenopsis* sp. in Taiwan (Jen et al., 2012). No nematode reproduction was observed, and the number of nematodes in the inoculated areas was very low. This study confirms the findings of previous work by Oliveira et al. (2019) on the diversity of *Aphelenchoides* species associated with strawberry in commercial Florida operations. Some of these species are facultative parasites that can reproduce on fungi but at the same time can parasitize and damage strawberry such as *A. besseyi*. Other species, such as *A. bicaudatus*, *A. pseudogoodeyi*, and *A. rutgersi* are mycetophagous. However, some of them, such as *A. bicaudatus* and *A. pseudogoodeyi*, can become facultative parasites behaving as phytoparasites under certain conditions.

Our study was limited to commercial strawberry fields in Florida; additional research on the species of *Aphelenchoides* associated with strawberry in operations outside Florida will provide a wider range of useful ecological information on the diversity of the *Aphelenchoides* species inhabiting the leaves of this industrial crop.
